# Polymerisation force of a rigid filament bundle: diffusive interaction leads to sublinear force-number scaling

**DOI:** 10.1038/s41598-018-20259-7

**Published:** 2018-02-06

**Authors:** Jemseena Valiyakath, Manoj Gopalakrishnan

**Affiliations:** 1 Department of Physics, Indian Institute of Technology Madras, Chennai, 600036 India; 2 Present Address: International Centre for Theoretical Sciences, Tata Institute of Fundamental Research, Bangalore, 560089 India

## Abstract

Polymerising filaments generate force against an obstacle, as in, e.g., microtubule-kinetochore interactions in the eukaryotic cell. Earlier studies of this problem have not included explicit three-dimensional monomer diffusion, and consequently, missed out on two important aspects: (i) the barrier, even when it is far from the polymers, affects free diffusion of monomers and reduces their adsorption at the tips, while (ii) parallel filaments could interact through the monomer density field (“diffusive coupling”), leading to negative interference between them. In our study, both these effects are included and their consequences investigated in detail. A mathematical treatment based on a set of continuum Fokker-Planck equations for combined filament-wall dynamics suggests that the barrier-induced monomer depletion reduces the growth velocity and also the stall force, while the total force produced by many filaments remains additive. However, Brownian dynamics simulations show that the linear force-number scaling holds only when the filaments are far apart; when they are arranged close together, forming a bundle, sublinear scaling of force with number appears, which could be attributed to diffusive interaction between the growing polymer tips.

## Introduction

The cytoskeleton, a network of filaments composed of actins and microtubules forms one type of force generators inside a eukaryotic cell and is crucial in moving cyotoplasmic material within the cell^1^. One manifestation of such force generation includes formation of the mitotic spindle, an apparatus formed by microtubules with associated molecular motors during cell division where the genetic material (chromosome pairs) are being pulled and pushed by microtubules until the sister chromatids are separated to each daughter nuclei^2,3^. Yet another instance is the migration of a cell from one place to another by crawling; in this case, polymerising actin filaments pushing the plasma membrane of the cell provides the mechanism for motility by generating cytosplasmic projections called filopodia and lamellipodia^4,5^. A number of mathematical and computational models have been proposed to study poymerisation-driven force generation in microtubules^6–13^ and actin^14–20^.

Outside the cytoplasmic environment, even a minimal system comprising of biofilaments growing against a barrier has been shown to be capable of generating forces of the order of a few piconewtons, for rigid^21–26^ as well as elastic^27–29^ barrier. For instance, a single microtubule grown *in vitro* can generate a maximum of 5pN force against a rigid barrier^21^. Microtubules growing against obstacles, tend to bend from their straight trajectory and then proceed to grow; a condition known as buckling^21,23^. Mechanically induced changes stemming from confinement are observed to alter the intrinsic dynamics of microtubules^23,30^. The catastrophe transition becomes pronounced in the vicinity of the barrier^30,31^, while the dynamic instability is observed to be regulated by force^23^.

It is well known that the physical barrier (the plasma membrane or proteinous kinetochore *in vivo*) in contact with the microtubule will affect the filament dynamics, by rendering steric hindrance to further polymerisation. Evidence for this scenario may be found in the reduced growth velocity near the cell boundary, as reported by^31^ in fission yeast cells; here, the reduction in growth velocity is speculated to be mechanical in origin. However, a slightly different explanation could be offered. Prior to the assembly, the monomers are diffusing in the cytoplasm or the available space of the experimental chamber, and any hindrance to free diffusion would have observable consequences; in particular, growth rate may be reduced due to hindrance to free diffusion offered by the barrier. This scenario appears to be partly supported by observations which find that growth of microtubule in the interior of the cell is different from near the boundary^32^. This effect is distinct from the steric hindrance to monomer addition, which comes into play only at extremely small barrier-tip separation (of the order of monomer length). A clear distinction between these two cases is not apparent from the existing experimental observations.

The origin of the polymerisation force is to be traced to the free energy change associated with polymerisation^33^. For example, the free energy released per GTP-tubulin addition to a microtubule is nearly 5–10 k_B_T, equivalent to a force of 50 pN if a microtubule grows by 8 nm; similarly the gain in free energy per GDP-tubulin dissociation from a microtubule is nearly 5–10 k_B_T^26^. However, experimental measurements suggest that the force produced by a microtubule is only a fraction of the maximum force predicted by theoretical arguments, suggesting that not all the free energy available from polymerisation of the 13 protofilaments is converted to work^21^. A similar phenomenon has been observed in the context of actin filaments polymerizing against load^34^; here, the force-velocity relation for a bundle was found to lie between the theoretical extremes corresponding to (a) perfect load-sharing and (b) no load-sharing between the filaments. Obtaining insights into this interesting nonlinear scaling behaviour is another important motivation for us in undertaking the present study.

**Figure 1:**
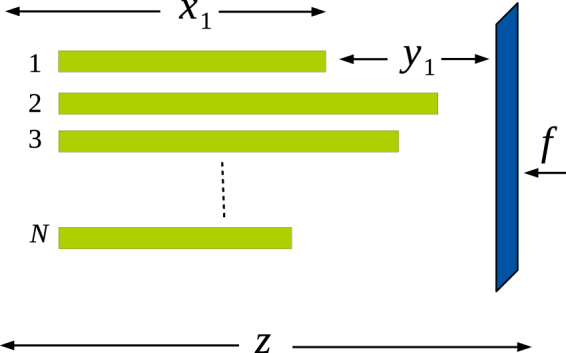
Schematic diagram for a bundle of inflexible filaments pushing against a movable rigid barrier acted upon by a constant force *f*. The rigid barrier also undergoes thermal motion characterised by diffusion coefficient *D*_*w*_.

Our model consists of a bundle of *N* rigid filaments (like microtubules) growing from one fixed wall of a compartment, towards the opposite wall, which is a diffusing barrier (diffusion coefficient *D*_*w*_), also being pushed backward with a constant force *f*, see Fig. 1. The filaments grow by diffusion-limited polymerisation of monomers, which are present in the solution at concentration *C*_0_. A filament also shrinks by random detachment of monomers, with rate *k*_off_. The filaments in the bundle are identical, and have equal base separation from each other. No interaction is assumed to exist between the filaments or between a filament and the barrier, except that neither of them can penetrate each other (see^12,13,15^ which explicitly considers the energy of interaction between the filaments). The filaments are assumed rigid, unaffected by thermal noise. We do not include the chemical switching activity of the monomers, e.g., hydrolysis of guanosine tri-phosphate (GTP) in microtubules, similar to some of the earlier studies^6,8,9,12^, our filaments are chemically inert.

The model is studied mathematically using Fokker-Planck equations and computationally using Brownian dynamics simulations. We present a systematic discussion of our methods in the next section, followed by results.

## Methods

### Mathematical formalism: Fokker-Planck equations

Most of the mathematical models that dealt with polymerisation-driven force generation do not explicitly consider the wall movements. Rather, the presence of wall is encapsulated in the growth rate and detachment rate of the filament^8–10,12,13^. In these ‘Brownian ratchet’ models, the filament in contact with the wall is assumed to grow with an on-rate proportional to exp (−*qfδ*/*k*_*B*_*T*) and off-rate proportional to exp (−(*q* − 1) *fδ*/*k*_*B*_*T*) with ‘*q*’ being the load sharing factor. Here, our approach is different; we adopt a formalism similar to that by Peskin *et al*.^6^. In this model, the wall executes a combination of diffusive (arising from thermal noise) and directed (due to the external force) one-dimensional motion. A continuum approximation is adopted for the filament dynamics as we find it more convenient to incorporate the continuous variation of on-rate with the wall-filament separation (discussed in more detail later).

The joint probability density function *P* (*X*, *z*; *t*) for the filament tip positions *X* ≡ {*x*_1_, *x*_2_, .., *x*_*N*_} and wall position *z*, in the continuum limit, satisfies the diffusion-drift equation

1 $$\frac{\partial P(X,z;t)}{\partial t}=-\frac{\partial }{\partial z}{J}_{w}(X,z;t)-\sum _{i=1}^{N}\frac{\partial }{\partial {x}_{i}}{J}_{i}(X,z,t).$$

Equation 1 is a Fokker-Planck equation in *N* + 1 variables, with both *x*_*i*_ and *z* lying in the interval (−∞, +∞). The individual probability currents corresponding to the dynamics of wall and filaments are given by

2 $${J}_{w}(X,z;t)={V}_{w}P(X,z;t)-{D}_{w}\frac{\partial }{\partial z}P(X,z;t),$$

3 $${J}_{i}(X,z;t)=V(z-{x}_{i})P(X,z;t)-\frac{\partial }{\partial {x}_{i}}[D(z-{x}_{i})P(X,z;t)].$$

In the above equation, *V*_*w*_ is the drift velocity of the wall in response to the external applied force *f*, and is related to its diffusion coefficient *D*_*w*_ through the Einstein-Smoluchowski relation *V*_*w*_ =−*fD*_*w*_/*k*_*B*_*T*^6^. The ‘diffusion coefficient’ *D* and ‘drift velocity’ *V* for the filament dynamics are expressed in terms of the on- and off-rates of monomers by the standard expressions^35^

4 $$D(z-{x}_{i})=[{k}_{{\rm{on}}}(z-{x}_{i})+{k}_{{\rm{off}}}]\frac{{\delta }^{2}}{2};\quad V(z-{x}_{i})=[{k}_{{\rm{on}}}(z-{x}_{i})-{k}_{{\rm{off}}}]\delta ,$$

where *k*_on_ (*z* − *x*_*i*_) is the position-dependent on-rate for monomer adsorption to the filament tip, the position dependence arising from the modification of the steady state concentration field due to the presence of the barrier (see Supplementary Information for details). *k*_off_, the off-rate of monomers is assumed to be a constant. We assume the presence of hard-core steric repulsion between the wall and the filaments; hence, the currents are subjected to reflecting boundary conditions at filament-wall contact:

5 $$\begin{array}{rcl}{{J}_{w}(X,z;t)|}_{z={x}_{i}} & = & 0\\ {{J}_{i}(X,z;t)|}_{{x}_{i}=z} & = & 0\end{array}\}\quad \forall i\in \mathrm{\ [1,}\,N\mathrm{].}$$

Let the instantaneous separation between the *i*^th^ filament and the wall be denoted

6 $${y}_{i}=z-{x}_{i},\quad \forall i\,\in \,[1,\,N]$$

which we shall refer to as ‘gaps’. Given the reflecting boundary conditions in Eq. 5, we expect the gap probability distribution (see Eq. 15 later) to become stationary for non-zero *f*, in the long-time limit. This conjecture helps us derive an expression for our main quantity of interest, i.e., the average filament/wall velocity, in a straightforward way. Considering this, we implement a change of variables in Eq. 1. All *x*_*i*_ are thus transformed into *y*_*i*_ by the relations given by Eq. 6. We denote the transformed probability density function as Π(*Y*, *z*; *t*), where *Y* = {*y*_1_, *y*_2_, ., *y*_*N*_}, hence Eq. 1 becomes,

7 $$\frac{\partial {\rm{\Pi }}(Y,z;t)}{\partial t}=-\frac{\partial }{\partial z}{\tilde{J}}_{w}(Y,z;t)-\sum _{i=1}^{N}\frac{\partial }{\partial {y}_{i}}{{\mathscr{K}}}_{i}(Y,z;t),$$

where, ${{\mathscr{K}}}_{i}(Y,z;t)\equiv {\tilde{J}}_{w}(Y,z;t)-{\tilde{J}}_{i}(Y,z;t)$. ${\mathop{J}\limits^{ \sim }}_{w}(Y,z;t)$ and ${\mathop{J}\limits^{ \sim }}_{i}(Y,z;t)$ are the transformed probability currents, in terms of the new variables, which are, respectively,

8 $${\tilde{J}}_{w}(Y,z;t)={V}_{w}{\rm{\Pi }}(Y,z;t)-{D}_{w}[\frac{\partial }{\partial z}+\sum _{i=1}^{N}\frac{\partial }{\partial {y}_{i}}]{\rm{\Pi }}(Y,z;t),$$

9 $${\tilde{J}}_{i}(Y,z;t)=V({y}_{i}){\rm{\Pi }}(Y,z;t)+\frac{\partial }{\partial {y}_{i}}[D({y}_{i}){\rm{\Pi }}(Y,z;t)].$$

${\tilde{J}}_{w}(Y,z;t)$ and ${{\mathscr{K}}}_{i}(Y,z;t)$ satisfy the boundary conditions (for 1 ≤ *i* ≤ *N*)

10 $${{\tilde{J}}_{w}(Y,z;t)|}_{z=\pm \infty }=0$$

11 $${{{\mathscr{K}}}_{i}(Y,z;t)|}_{{y}_{i}=0}=0;\quad \quad {{{\mathscr{K}}}_{i}(Y,z;t)|}_{{y}_{i}=\infty }=0.$$

The average position of the wall is given by

12 $$\bar{z}(t)={\int }_{-\infty }^{\infty }{\int }_{0}^{\infty }{\rm{\Pi }}(Y,z;t)zdzdY.$$

Applying Eq. 12 in Eq. 7 and using the boundary condition given by Eqs 10 and 11, we arrive at the following expression for the mean wall velocity

13 $${V}_{N}(f)\equiv \frac{d\bar{z}}{dt}={V}_{w}+N{D}_{w}{\varphi }_{N}(0,t),$$

where *ϕ*_*N*_ (*y*, *t*) is the probability density for the separation *y* between the wall and one of the filaments (single filament gap size distribution), i.e.,

14 $${\varphi }_{N}(y,t)=\int {\rm{\Phi }}(y,{y}_{2},\mathrm{...}{y}_{N};t)d{y}_{2}\mathrm{...}d{y}_{N}.$$

The integrand Φ(*Y*; *t*), gives the joint probability distribution of gap lengths:

15 $${\rm{\Phi }}(Y;t)=\int {\rm{\Pi }}(Y,z;t)dz.$$

From Eq. 6, it follows that in the long time limit (where *y*_*i*_ are expected to become stationary variables), the average wall velocity becomes equal to the average filament velocity, hence it is sufficient to get an expression for the wall velocity using Eq. 13, using which one can study the wall-induced effects on the kinetics of polymerisation and force generation.

### Brownian dynamics simulations

**Figure 2:**
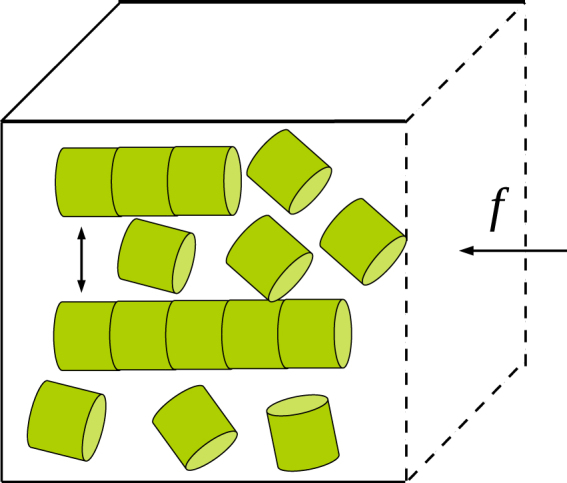
Schematic diagram of the cubical box, containing a bundle of filaments growing by a diffusion-limited reaction used in Brownian dynamics simulations. One face of the cubical box facing the filament tip is movable (the barrier); it is acted on by a constant force *f* in the backward direction and also undergoes random motion characterised by diffusion coefficient *D*_*w*_.

The mathematical formalism presented earlier has two limitations: (i) diffusion of monomers is not taken into account explicitly, rather, it enters through the gap-dependent on-rate of monomers (ii) the length of the polymers is treated as a continuous variable, ignoring the discreteness of monomer addition and dissociation processes. To overcome these limitations, we also carried out Brownian dynamics simulations; here, the free monomers are treated as point particles, and diffuse inside a rectangular box, with the walls of the box acting as reflecting boundaries, see Fig. 2.

The positions of the individual monomers **r**_*m*_ (*t*) and the wall (movable face of the rectangular box) are updated using overdamped Langevin equations. Over a small time step Δ*t*, the updating rules have the form,

16 $$\begin{array}{c}{{\bf{r}}}_{m}(t+{\rm{\Delta }}t)={{\bf{r}}}_{m}(t)+\sqrt{6D{\rm{\Delta }}t}\,\eta ,\\ z(t+{\rm{\Delta }}t)=z(t)-\frac{f{D}_{w}}{{k}_{B}T}{\rm{\Delta }}t+\sqrt{2{D}_{w}{\rm{\Delta }}t}\,{\eta }_{w},\end{array}$$

where *η* = (*η*_*x*_, *η*_*y*_, *η*_*z*_), the latter being random numbers drawn from independent Gaussian distributions with zero mean and unit variance. Similarly, *η*_*w*_ is a Gaussian random variable with zero mean and unit variance. We used Δ*t* = 10^−4^ s, *D*_*w*_ = 10^3^ nm^2^ s^−1^ and *D* = 10^5^ nm^2^ s^−1^; for diffusing monomers, this implies a mean free path $\sim \sqrt{D{\rm{\Delta }}t}\simeq 3$ nm between successive changes in direction. A ratio of 100:1 was kept between the diffusion coefficients of the wall and the monomers since the objects that obstruct the free growth of microtubule as well as free diffusion of tubulins include the lipid membranes or a kinetochore, which are massive compared to tubulin monomers. Three boundary conditions are imposed on the diffusing monomers, (i) reflecting boundary conditions on the walls of the rectangular box, (ii) reflecting boundary condition on the cylindrical wall of the filament and (iii) absorbing boundary condition at the circular face/tip of the filament. These boundary condtions are implemented as follows: if a predicted increment vector in the position of a monomer *r*_*m*_ (*t* + Δ*t*) − r_*m*_ (*t*) during a time interval Δ*t* crosses either an outer wall or the cylindrical surface of a filament, it is “reflected” at the point of contact about the normal to the surface (similar to a light ray) and the monomer’s path is modified (such that the total absolute distance covered during Δ*t* is the same as what it would have been without reflection). For an absorbing surface, the monomer vanishes upon making contact with the surface. Although the monomers are treated as point particles when simulating their diffusion, once a monomer is adsorbed onto a polymer tip, the length of the polymer increases by *δ* (however, this requirement was waived in one set of simulations, see (i) below). The initial spatial distribution of free monomers is uniform, with concentration *C*_0_. In order to ensure that adsorption events at polymer tips do not cause depletion in the *total* number of monomers, every time a monomer disappeared from the solution by binding to a polymer tip, a new monomer was added at a random location inside the box. This procedure ensures that the free monomer concentration far from the absorbing tips is always *C*_0_. In the simulations, we analyzed three different cases and are summarized below.

(i) *On-rate of monomers binding to static disc-shaped absorbing surface*
  In the first set of simulations, we looked at the steady state adsorption of particles to a static filament (filament length remains the same irrespective of monomer adsorption) with all the faces of the box kept fixed, by varying the separation between the filament tip and the face opposite to it. Here, the filament as well as the wall are static in space.
(ii) *FFD filaments growing against a mobile barrier*
  In the second set of simulations, we studied the force-velocity relation for rigid linear polymers with monomeric units modeled as flat-faced discs of different radii of cross section. In addition, we also varied the base separation between the filaments. Unlike the earlier case, here, whenever a monomer is adsorbed, the length of the filament increases by *δ*; the mean length of a filament grows linearly with time. We also allow a bound monomer to occasionally detach from the filament after adsorption, and this is accounted for in the simulations by including a non-zero off-rate. Our simulations are done at fixed *mean* monomer concentration *C*_0_ (as explained earlier). The mean velocity of growth of a filament was measured as the slope of the graph of the mean length versus time, with the averaging done over 1000 independent runs. For different values of *f*, we calculated the average velocity of growth of the filament, for *a* = 20 nm, 10 nm and 2 nm, with *δ* = 2 nm in all the three cases. The mean velocity was plotted as a function of the force *f*; the stall force *f*_*s*_, the point of zero-crossing of the *V* − *f* curve, was determined by linear interpolation.
(iii) *Multi-stranded polymers with microtubule-like geometry*

**Figure 3:**
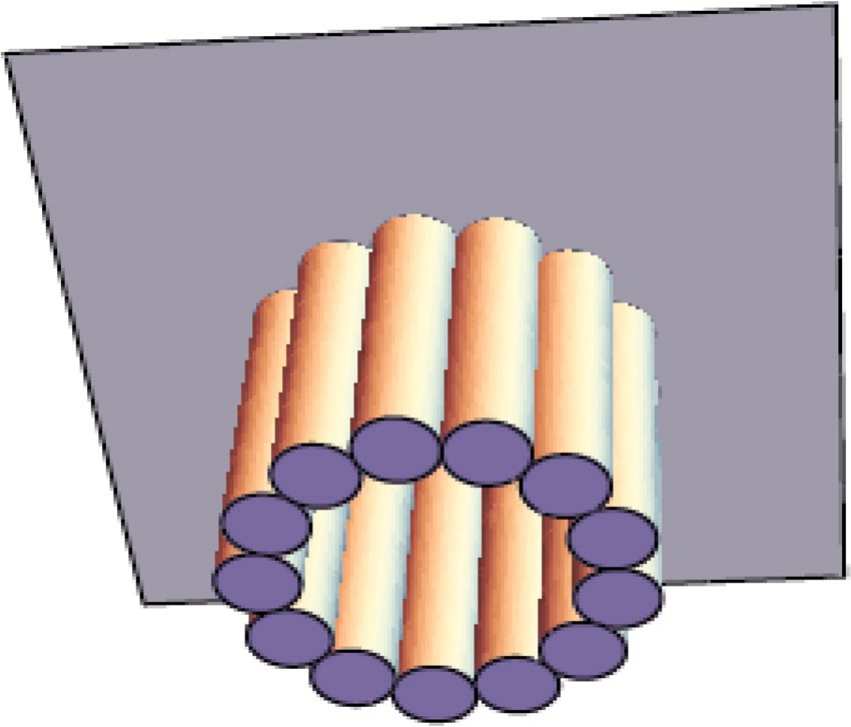
A schematic diagram of a multi-stranded filament with microtubule-like geometry.

In the next stage, we extended our simulations to multi-stranded polymers with microtubule-like geometry (but without hydrolysis or dynamic instability, see the schematic, Fig. 3). Here, each polymer consists of 13 protofilaments (with each protofilament being a FFD polymer with radius *a* = 2.5 nm, similar to one of the cases studied in (ii)), arranged in a circular fashion, with outer radius 12.5 nm and inner radius 7.5 nm. Each protofilament here grows and shrinks individually, with diffusion-limited binding and random detachment (off-rate *k*_off_) of monomers. The monomers here are circular discs of radius 2.5 nm and length *δ* = 8 nm. To calculate the mean velocity of growth, we tracked the time evolution of the length of one randomly chosen protofilament belonging to one of the microtubules (if there are more than one) in one simulation. The results are averaged over 500 independent runs.

## Results

### Mathematical results: Gap distribution and mean filament velocity

To derive an expression for the mean velocity of the filament/wall, we first need to find the expression for the single filament gap distribution, *ϕ*_*N*_ (*y*), *t*. After integrating over *y*_2_, ...*y*_*N*_, *z* in the general equation for Π (*Y*, *z*; *t*) given by Eq. 7, using the boundary conditions for ${\tilde{J}}_{w}(Y,z;t)$ and ${{\mathscr{K}}}_{i}(Y,z;t)$ given by Eqs 10–11 we find

17 $$\frac{\partial {\varphi }_{N}(y,t)}{\partial t}=-\frac{\partial }{\partial y}\bar{{\mathscr{K}}}(y,t)$$

where

18 $$\bar{{\mathscr{K}}}(y,t)=\int {{\mathscr{K}}}_{i}(y,{y}_{2},\mathrm{...}{y}_{N},z;t)d{y}_{2}\mathrm{...}d{y}_{N}dz;\quad {\bar{{\mathscr{K}}}(y,t)|}_{y=0,\infty }=0$$

is an effective single-filament probability current density, the boundary conditions follow from Eqs 10 and 11 upon integration. In the long-time limit, for *f* > 0, the distribution *ϕ*_*N*_ (*y*, *t*) is expected to become stationary: denote *ϕ*_*N*_ (*y*, *t* → ∞) ≡ *ϕ*_*N*_ (*y*) and $\bar{{\mathscr{K}}}(y,t\to \infty )\equiv \bar{{\mathscr{K}}}(y)$; hence the L.H.S of Eq. 17 can be put to zero to give $\partial \bar{{\mathscr{K}}}(y)/\partial y=0$, which, when combined with the boundary conditions in Eq. 18, yields the important relation

19 $$\bar{{\mathscr{K}}}(y)=0$$

Implementing this condition in Eq. 18 and using the defining relations in Eqs 7–11, we find that *ϕ*_*N*_ (*y*) satisfies the equation

20 $$[V(y)-{V}_{w}]{\varphi }_{N}(y)+\frac{\partial }{\partial y}[D(y)+{D}_{w}]{\varphi }_{N}(y)-(N-1){D}_{w}{F}_{N}(y)=0,$$

where

21 $$\begin{array}{c}{F}_{N}(y)=\int \Phi (y,{y}_{2}=0,{y}_{3},\mathrm{..},{y}_{N})d{y}_{3}\mathrm{..}d{y}_{N}\quad N\ge 3,\\ \quad \quad \quad \quad \quad \quad \quad \quad \quad \quad \quad {F}_{2}(y)={\rm{\Phi }}(y,{y}_{2}=0),\end{array}$$

such that

22 $${\int }_{0}^{\infty }{F}_{N}(y)dy={\varphi }_{N}(0).$$

The formal solution to Eq. 20 can be written as

23 $${\varphi }_{N}(y)=\frac{{e}^{-\int {\rm{\Gamma }}(y^{\prime} )dy^{\prime} }}{D(y)+{D}_{w}}[{\mathscr{A}}+{D}_{w}(N-1){\int }^{y}dy^{\prime} {e}^{\int {\rm{\Gamma }}(y^{\prime\prime} )dy^{\prime\prime} }{F}_{N}(y^{\prime} )],$$

with

24 $${\rm{\Gamma }}(y)=\frac{V(y)-{V}_{w}}{D(y)+{D}_{w}}.$$

In order to calculate *ϕ*_*N*_ (*y*), we use the following mathematical forms for *V* (*y*) and *D* (*y*), obtained using the approximate position-dependent on-rate ${k}_{{\rm{on}}}\simeq {k}_{{\rm{on}}}(\infty \mathrm{)[1}-{e}^{-\lambda y}]$ (see Supplementary Information) for FFD polymers. Here, we expect from scaling considerations that $\lambda \sim 1/a$, where *a* is the radius of the disc. It then follows from Eq. 4 that

25 $$V(y)={V}_{0}-{V}_{1}{e}^{-\lambda y};\quad \quad D(y)={D}_{0}-{D}_{1}{e}^{-\lambda y},$$

where

26 $${V}_{0}=[{k}_{{\rm{o}}{\rm{n}}}({\rm{\infty }})-{k}_{{\rm{o}}{\rm{f}}{\rm{f}}}]\delta ;\quad {V}_{1}={k}_{{\rm{o}}{\rm{n}}}({\rm{\infty }})\delta .$$

Similarly,

27 $${D}_{0}=[{k}_{on}(\infty )+{k}_{{\rm{off}}}]\frac{{\delta }^{2}}{2};\quad {D}_{1}={k}_{{\rm{on}}}(\infty )\frac{{\delta }^{2}}{2}.$$

For *N* = 1, the gap size distribution is given by

28 $${\varphi }_{1}(y)={\mathscr{A}}{e}^{-{Q}_{2}y}{[1-{\mathscr{D}}{e}^{-\lambda y}]}^{-{P}_{2}}$$

where,

29 $${Q}_{2}=\frac{{V}_{0}-{V}_{w}}{D^{\prime} };\quad {P}_{2}=[1+\frac{{V}_{0}-{V}_{w}}{\lambda D^{\prime} }-\frac{{V}_{1}}{\lambda {D}_{1}}];{\mathscr{D}}=\frac{{D}_{1}}{D^{\prime} }\quad D^{\prime} ={D}_{0}+{D}_{w}\mathrm{.}$$

The normalization constant ${\mathscr{A}}$ is given by

30 $${\mathscr{A}}={[\sum _{n=0}^{\infty }\frac{{({P}_{2})}_{n}{{\mathscr{D}}}^{n}}{n!}\frac{1}{[{Q}_{2}+n\lambda ]}]}^{-1}.$$

The corresponding results for *N* = 2 are given in the Supplementary Information.

### Special case: *λ* → ∞ (constant on-rate) and general *N*

The simplest limit, corresponding to *λ* = ∞, is similar to the studies by Peskin *et al*.^6^; here, we have

31 $$V(y)\equiv {V}_{0}=[{k}_{{\rm{on}}}(\infty )-{k}_{{\rm{off}}}]\delta ;\quad D(y)\equiv {D}_{0}=[{k}_{{\rm{on}}}(\infty )+{k}_{{\rm{off}}}]\frac{{\delta }^{2}}{2}.$$

In this limit, the single filament gap distribution (Eq. 23) is given by

32 $${\varphi }_{N}(y)=\frac{{e}^{-{{\rm{\Delta }}}_{1}y}}{{D}_{0}+{D}_{w}}\{{\mathscr{A}}+{D}_{w}(N-1){\int }^{y}dy^{\prime} {e}^{{{\rm{\Delta }}}_{1}y^{\prime} }{F}_{N}(y^{\prime} )\},$$

with

33 $${{\rm{\Delta }}}_{1}=\frac{{V}_{0}-{V}_{w}}{{D}_{0}+{D}_{w}};$$

The expression for *F*_*N*_ (*y*) is a simple exponential here:

34 $${F}_{N}(y)= {\mathcal B} {e}^{-{{\rm{\Delta }}}_{2}y},$$

where

35 $${{\rm{\Delta }}}_{2}=\frac{{V}_{0}-{V}_{w}}{{D}_{0}+N{D}_{w}}.$$

Now, substituting Eqs 32 and 34 in Eq. S40 given in the Supplementary Information, we get

36 $${\mathscr{A}}= {\mathcal B} \,[\frac{{D}_{0}+{D}_{w}}{{{\rm{\Delta }}}_{2}}-\frac{(N-1){D}_{w}}{{{\rm{\Delta }}}_{1}-{{\rm{\Delta }}}_{2}}].$$

On using the normalization condition (Eq. S39 in the Supplementary Information) in Eq. 32 we find that

37 $$ {\mathcal B} ={{\rm{\Delta }}}_{1}{{\rm{\Delta }}}_{2}{[1+\frac{(N-1){D}_{w}}{{D}_{0}+{D}_{w}}]}^{-1}.$$

Substituting Eq. 34 with ${\mathcal B}$ given by Eq. 37 in Eq. 32 and performing the integration we get

38 $${\varphi }_{N}(y)={{\rm{\Delta }}}_{2}{e}^{-{{\rm{\Delta }}}_{2}y}.$$

Substitution of *ϕ*_*N*_ (0) = Δ_2_, as calculated using Eq. 38, in the general expression for the average velocity given by Eq. 13 gives

39 $${V}_{N}(f)=\frac{{V}_{w}{D}_{0}+N{D}_{w}{V}_{0}}{{D}_{0}+N{D}_{w}}\quad (\lambda =\infty ).$$

The stall force, corresponding to zero mean velocity, is obtained by putting *V*_*N*_ (*f*) = 0 in Eq. 39, and is given as

40 $${f}_{s}^{N}=\frac{2N{k}_{B}T}{\delta }[\frac{{k}_{{\rm{on}}}(\infty )-{k}_{{\rm{off}}}}{{k}_{{\rm{on}}}(\infty )+{k}_{{\rm{off}}}}]\quad (\mathrm{continuum}\,\mathrm{model})\mathrm{.}$$

For constant on-rate case, the stall force scales linearly with the number of filaments, similar to earlier predictions^6,9,36^. But the mathematical dependence of stall force on the on-rate and off-rate differ, the difference clearly arising from the continuum treatment in this paper as opposed to the discrete approach in van Doorn *et al*.^9^. The corresponding prediction of the discrete model^6,9,36^ is

41 $${f}_{s}^{N}=\frac{N{k}_{B}T}{\delta }\,\mathrm{ln}[\frac{{k}_{{\rm{on}}}(\infty )}{{k}_{{\rm{off}}}}]\quad (\mathrm{discrete}\,\mathrm{model})\mathrm{.}$$

Note that *f*_*s*_ (*N*) = 0 in both Eqs 40 and 41 when *k*_on_ = *k*_off_. The latter corresponds to the state of chemical equilibrium of the system, where growth and detachment processes balance each other on average (with corresponding drop in the free monomer concentration in solution), and there is no net growth for the polymer (hence no more work can be extracted). It is also easily verified that in the limit *k*_on_ (∞) ≈ *k*_off_, Eq. 41 agrees with Eq. 40, which is to be expected, as a continuum approximation works best when the (length) increment per unit step is small.

In Supplementary Information, we show that the linear scaling of stall force with number holds true to ${\mathscr{O}}\mathrm{(1/}\lambda )$, although both the filament velocity and the stall force are found to be reduced. These predictions are subjected to further examination in the following subsection.

### Simulation results

#### Static “absorbing” disc

In the first set of simulations we studied the on-rate of monomer adsorption to a static surface, in the presence of a reflecting wall, as mentioned in case (i) of Sec. 1, for various radii of cross-section of the circular absorbing surface. From the simulation results, it is observed that the on-rate of monomers is dependent on the separation between the surface and the wall; as the separation decreases, a substantial drop in the on-rate is seen. The data along with the analytical results is discussed in detail in Supplementary Information, and was used in analytical calculations in the previous subsection.

#### FFD filaments

**Figure 4:**
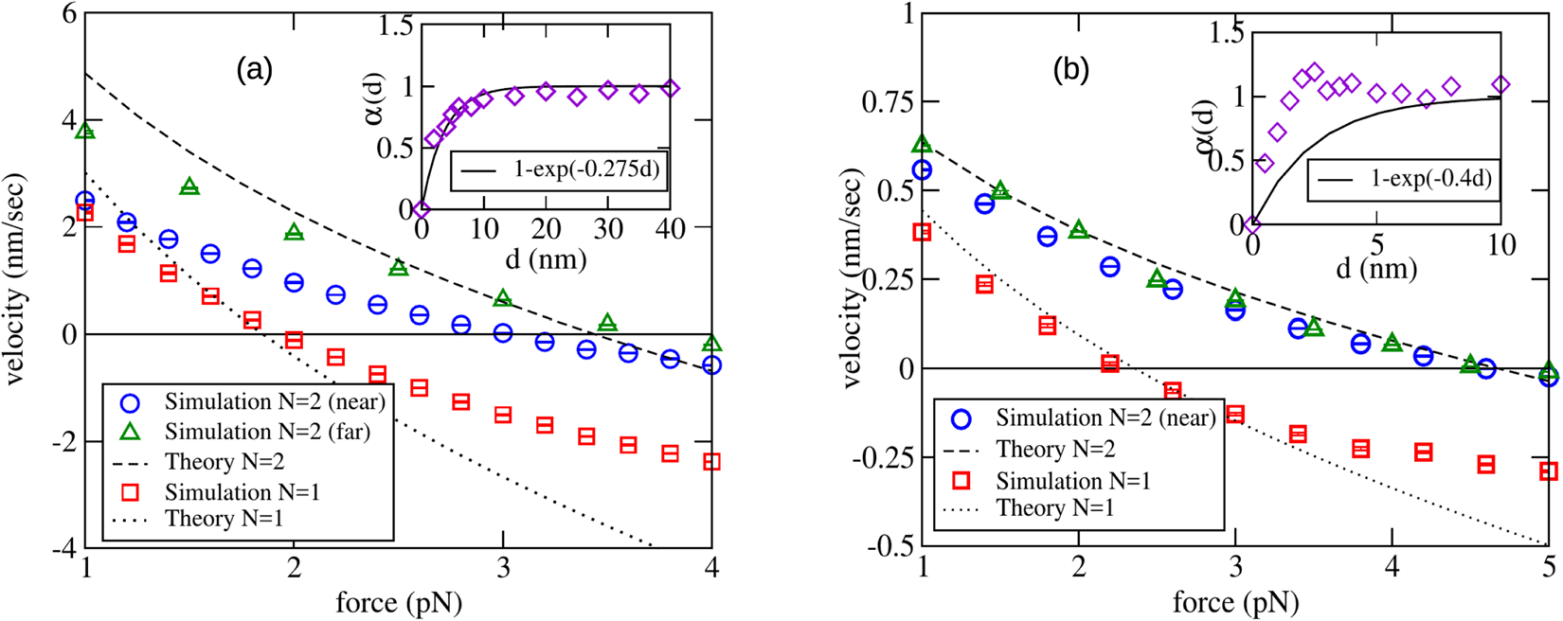
The force-velocity curve for a single filament versus two filaments, obtained from Brownian dynamics simulations, for (**a**) *a* = 10 nm and (**b**) *a* = 2 nm. For (**a**), the off-rate of monomers is *k*_off_ = 2 s^−1^ while for (**b**), *k*_off_ = 0.2 s^−1^. Analytical results are shown for best fit value of *λ*; 0.275 nm^−1^ in (**a**) and 0.4 nm^−1^ in (**b**). In the insets, fits for (scaled) separation-dependent on-rate *α* (*d*) = *k*_on_ (*d*)/*k*_on_ (∞), using the same *λ* are shown. The other parameter values are given in Table 1. Here, *near* means zero base separation between polymers, wheras *far* refers to a base separation 10*a*. The error bars are typically smaller than the size of the symbols.

In the second set of simulations, we studied the force-velocity relation for FFD filaments. The various parameters are summarised in Table 1. Figure 4a shows the data for *a* = 10 nm, for one and two filament systems. In the second case, two values for the lateral base separation between filaments was studied; 0 and 100 nm (here, zero base separation refers to the filaments touching each other). The two cases are distinguished in the plots as “near” and “far”. The stall force for a single filament is found to be $\simeq 1.94$ pN. By comparison, Eq. 41 predicts a stall force of $\simeq 2.62$ pN. The discrepancy is almost certainly arising from the barrier-induced reduction in the on-rate, which is significant for *a* = 10 nm. The inset of the same Figure shows the dimensionless on-rate for monomer binding onto an absorbing disc of the same radius, as a function of its distance from a reflecting barrier. Fitting the data to an exponential curve yields the parameter *λ*, which is then used to predict the force-velocity relation using the relevant equations from Sec. 3, viz., Eqs 28 and 13 (for *N* = 1) and Eq. S43 in the Supplementary Information (for *N* = 2). The theoretical predictions are also shown alongside the simulation results in the same Figure. For *N* = 1, the agreement between the theoretical curve and simulation data is excellent in the sub-stall regime, while significant deviation is observed post-stall. For *N* = 2, the far-data shows reasonable agreement with the theoretical curve except at very small *f*, but the near-data is significantly different. More importantly, while the two-filament stall force ${f}_{s}^{(2)}\simeq 2{f}_{s}^{(1)}$ for filaments far apart, ${f}_{s}^{(2)} < 2{f}_{s}^{(1)}$ for near-filaments, i.e., sublinear scaling of stall force with number is observed when the filaments are close together, but linear scaling is restored when they are far apart. This observation is in disagreement with the prediction of the Brownian ratchet model^6^. We strongly believe that the sublinear scaling arises from *diffusive interaction* between the filaments, a phenomenon that occurs whenever multiple ‘sinks’ compete for diffusing particles that form a common pool^37–39^.

**Table 1:** Numerical values of the various parameters used in the Brownian dynamics simulation for flat-faced filaments (FFD) and microtubule-like geometry.

Parameter	Symbol	FFD	MT-like geometry
Monomer length	*δ*	2 nm	8 nm
Radius	*a*	variable	2.5 nm
Diffusion coefficient (monomer)	*D*	10^5^ nm^2^ s^−1^	10^5^ nm^2^ s^−1^
Diffusion coefficient (wall)	*D*_*w*_	10^3^ nm^2^ s^−1^	10^3^ nm^2^ s^−1^
Concentration	*C*_0_	3.3 *μ*M	33.3 *μ*M
Monomer dissociation rate	*k*_off_	variable	3 s^−1^

We repeated the above investigations for a smaller radius of cross-section, *a* = 2 nm. The results are shown in Fig. 4b. The observed single filament stall force here is nearly 2.27 pN, while the theoretical prediction from Eq. 41 of the discrete model is $\simeq 2.6$ pN. The velocity-force curves predicted using the continuum model also agree with the simulations over a larger range of force. Coming now to two-filament data, unlike the previous case, the near and far cases for *N* = 2 are practically indistinguishable here, and both agree very well with the theoretical curve (here, “far” refers to a base separation of 20 nm). Finally, the two-filament stall force is very nearly twice the single filament force, indicating that diffusive interaction is negligible here, at least for *N* = 2. However, we shall see in the next subsection that for larger numbers, this interaction becomes significant even for *a* ~ 2 nm. For two filaments, the observed doubling of stall force for *N* = 2, when the filaments are far apart, is also consistent with the results of the asymptotic analysis (*λ* → ∞) presented in Supplementary Information.

**Figure 5:**
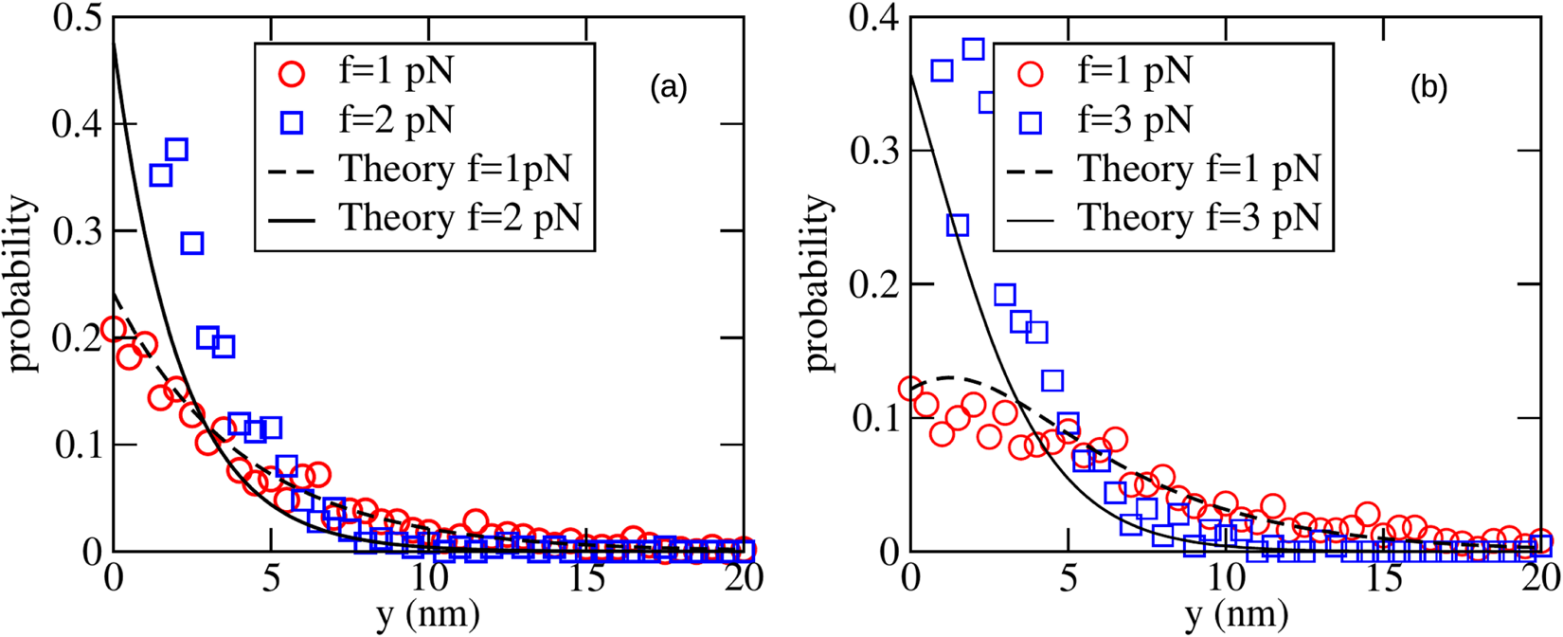
The gap distribution for *a* = 10 nm, for two forces, far from and near to stall, with (**a**) *N* = 1 and (**b**) *N* = 2. Fits of the analytical results, Eq. 28 for *N* = 1 and Eq. S43(Supplementary Information) for *N* = 2 are also shown for the best fit value *λ* = 0.275 nm^−1^, with *k*_on_ (∞) = 7.5 s^−1^. For both (**a**) and (**b**), *k*_off_ = 2 s^−1^. The other parameter values are listed in Table 1.

**Figure 6:**
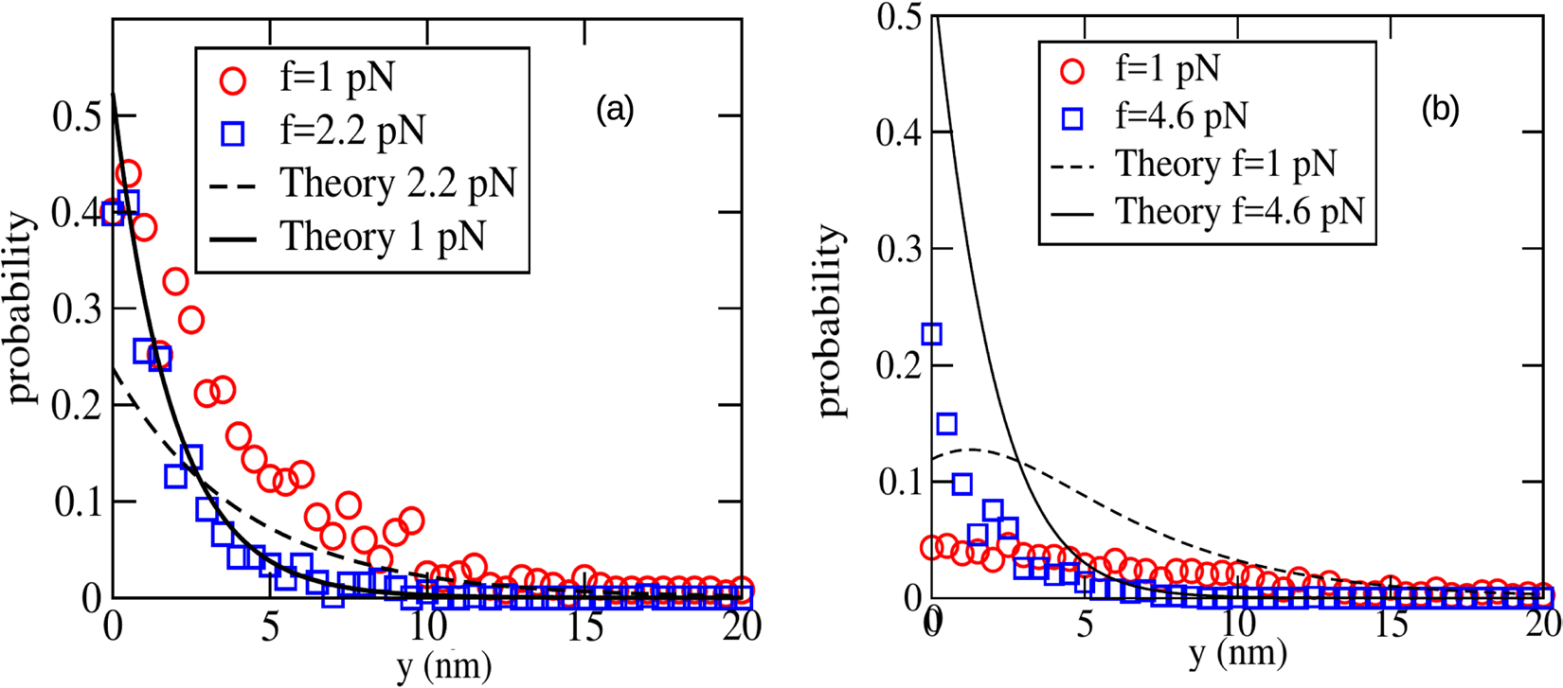
The gap distribution for *a* = 2 nm, for two forces, far and near to stall. (**a**) is shown for *N* = 1 and (**b**) is shown for *N* = 2. A fit of the analytical results (Eq. 28 for *N* = 1 and Eq. S43(Supplementary Information) for *N* = 2) also shown for the best fitting value of *λ* = 0.4 nm^−1^, with *k*_on_ (∞) = 0.73 s^−1^. For both (**a**) and (**b**), *k*_off_ = 0.2 s^−1^. The other parameter values are listed in Table 1.

As further verification of the continuum theory presented in the last section, we also found the single-filament gap distribution function *ϕ*_*N*_ (*y*) (defined in Eq. 14), and compared with the theoretical predictions given in Eq. 28 (*N* = 1) and Eq. S43 in the Supplementary Information (*N* = 2). The results are given in Fig. 5 (*a* = 10 nm) and Fig. 6 (*a* = 2 nm). Quantitative agreement is better for *a* = 10 nm compared to *a* = 2 nm, as expected.

#### Microtubule-like filaments

**Figure 7:**
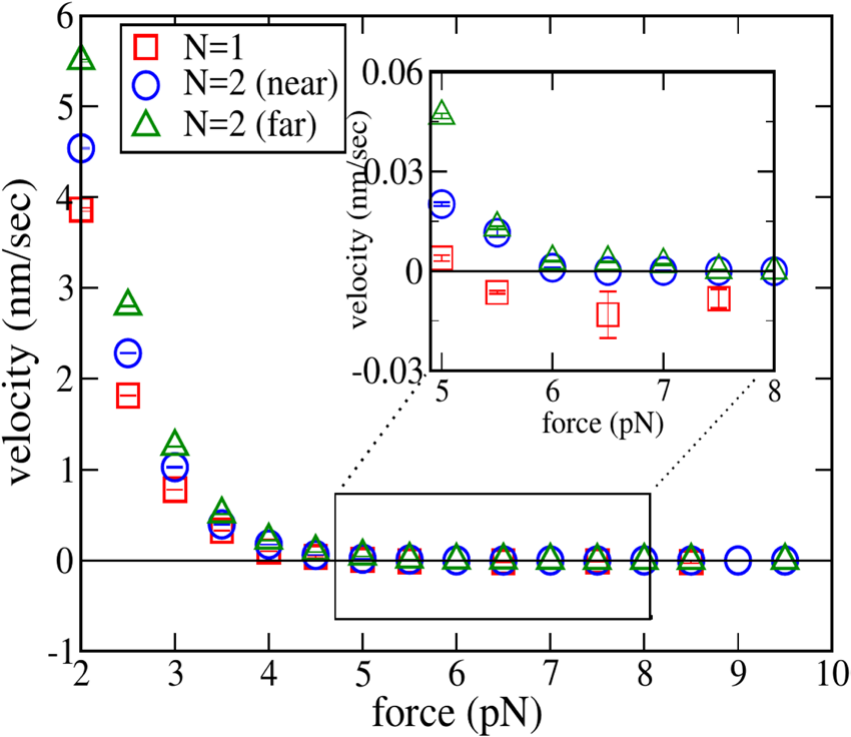
Force-velocity relation for a single microtubule and two microtubules, both *near* (zero base separation) and *far* (base separation of 150 nm). The values of the parameters used in the simulation are listed in Table 1. The inset zooms the force range where the velocity vanishes. Note that while the single filament curve crosses the *x*-axis after touching zero at stall, the two-filament velocity remains close to zero after stall. In most cases, the error bars are smaller than the size of the symbols. For two filaments, ‘near’ means zero base separation, while ‘far’ refers to a base separation 150 nm.

Figure 7 shows the force-velocity curve for one (*N* = 1) and two (*N* = 2) microtubule-like filaments. The observed stall force for a single microtubule is found to be nearly 5.17 pN in simulations for the parameters used here. For comparison, for the same set of parameters, the prediction of the Brownian ratchet model (Eq. 41) for the combined stall force of 13 independent protofilaments is 12.24 pN. Therefore, we again encounter sublinear scaling of stall force, here as a function of the number of (proto)filaments, similar to the experimental observations in^21^. This can be explained using two different, but essentially equivalent arguments:

- a protofilament here is part of the larger microtubule, which has an outer radius of nearly 12.5 nm, large enough for significant barrier-induced reduction in the on-rate, when the filament is close enough to the wall. This causes each protofilament to grow much slower than it would have, if it were alone in the solution. Consequently growth is stalled at a lower value of the opposing force.
- each protofilament is diffusively coupled to the other protofilaments, and hence the on-rate for one is reduced by the presence of the others. For *n* disc-shaped absorbers (each with radius *a*) arranged uniformly in a circle, with centre-to-centre separation *R*, it has been proposed that, for $R\gg a$, the effective diffusion-limited on-rate for one of the discs is given by the approximate formula^40^

42 $${k}_{{\rm{on}}}\simeq \frac{4Da{C}_{0}}{1+\frac{2a}{\pi R}\,\mathrm{ln}(\frac{2n}{\pi })}\quad \quad \quad R\gg a$$

**Figure 8:**
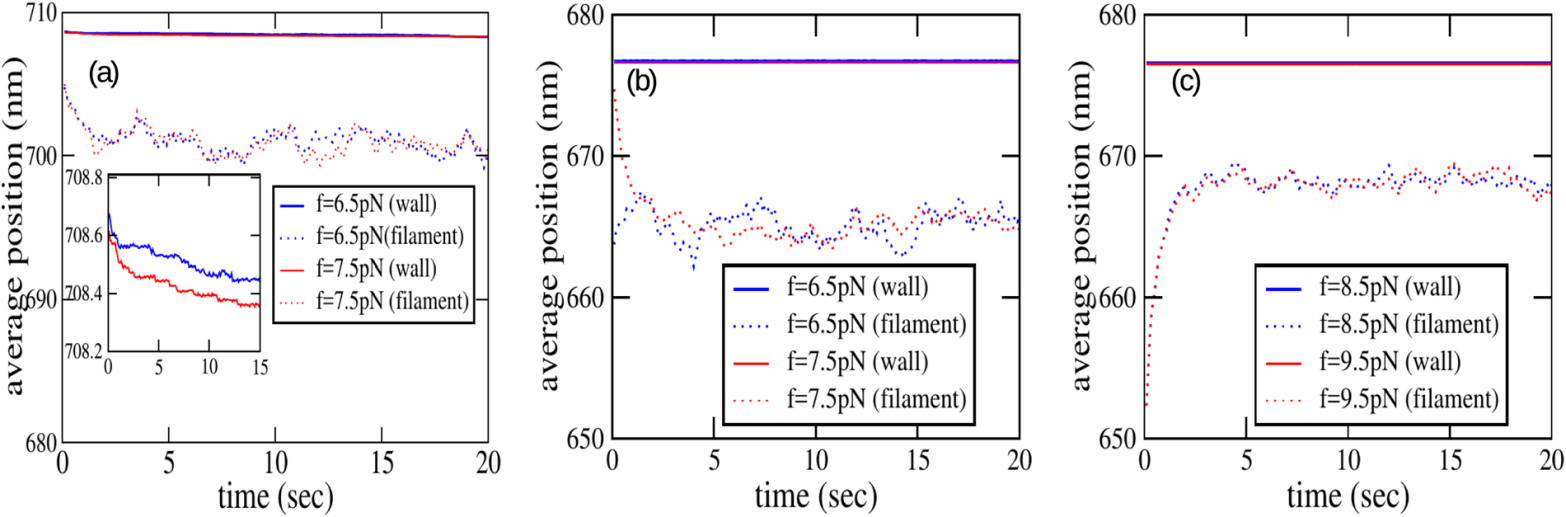
The time evolution of the average positions of the wall and one of the protofilaments is shown for (**a**) one microtubule, (**b**) two microtubules (near) and (**c**) two microtubules (far). In the inset of (**a**), the average position of wall alone shown. The parameters common for all the three cases are listed in Table 1. For two filaments, ‘near’ means zero base separation, while ‘far’ refers to a base separation 150 nm.

In a microtubule, protofilaments are tightly packed, this corresponds to the situation with $R\simeq 2a$ in the above formula. However, protofilament lengths can be different in general, hence it is not clear *a priori* if Eq. 42 can be applied in this case. Nevertheless, it is remarkable that the stall force of a single microtubule calculated using Eq. 41, with the on-rate given by Eq. 42 (after substituting *a* = 2.5 nm, *R* = 2*a*, *n* = 13 and *δ* = 8 nm), turns out to be 3.94 pN, closer to the observed value. The velocity-force curves of two microtubule-like filaments, both *near* (base separation zero) and *far* (base separation 150 nm), show a surprising feature. A close inspection (see inset of Fig. 7) reveals that the two-filament mean velocity remains close to zero after reaching stall (|*V*_2_(*f*)| < 10^−3^ nm s^−1^ for $f\ge {f}_{s}^{(2)}$). This counter-intuitive behaviour persists for forces up to 10 pN; the growth velocity remains close to zero for a large range of force in the super-stall regime. At present, we do not have an explanation for this observation. Nevertheless, Fig. 8 provides some insights. Here, we show comparisons of the time-dependence of the mean position of the barrier and a randomly chosen protofilament for *N* = 1 (a) and *N* = 2 (b and c), at *super-stall* forces. For *N* = 1, the wall and the filament keeps moves leftward on average, keeping a constant mean separation between them. Something different happens for *N* = 2. Here, as the force is increased, the mean positions of both the filament tip and the wall shifts leftwards, but settles in a new equilibrium position, with a constant, force-dependent mean separation between the two (Fig. 8). Since both near and far configurations show similar qualitative behaviour, it appears that the large number of individual (proto)filaments for *N* = 2 (26 in total) might be the crucial factor here; this issue requires further investigation. It is also likely that these new “equilibrium” states may be actually metastable; such metastable states have been revealed in a recent investigation of the growth dynamics of parallel actin bundles^41^.

The sub-additive nature of the stall force of a single microtubule reported in the experiments^21^ was also investigated theoretically by Mogilner and Oster^8^. Their argument was that, because of variations in the individual protofilament lengths, the mean number of protofilaments in contact with the barrier is less than the total number, and this leads to sublinear scaling of force with number. However, this argument was refuted by van Doorn *et al*.^9^, who showed that the conclusion of Mogilner and Oster^7^ was an artifact of their deterministic, continuum approximation scheme, and that linear scaling of force with number is recovered when the discrete stochastic formalism is used. We show here that the sublinear scaling of stall force of a microtubule is a reality, and arises from diffusive interaction between growing filament tips, an effect not taken into account in van Doorn *et al*.^9^.

The deviation from linear scaling of the stall force may be characterised in simulations using a scaling parameter $\nu ={f}_{s}^{(2)}/2{f}_{s}^{(1)}$, which is always 1 for perfect linear scaling. In Table 2, we collect together the different values of stall forces observed in our simulations, as well as the computed *ν*, for FFD filaments and multi-stranded microtubule-like filaments.

**Table 2:** The stall forces for single and two filaments, and the scaling parameter $\nu ={f}_{s}^{2}/2{f}_{s}^{1}$, for *N* = 1 and 2, for flat-faced disc polymers and microtubule-like polymers.

Filament	${f}_{s}^{1}$(pN)	${f}_{s}^{2}$(pN)	*ν*
FFD (*a* = 2 nm, near)	2.27 ± 0.05	4.58 ± 0.05	1.01 ± 0.022
FFD (*a* = 2 nm, far)	2.27 ± 0.05	4.53 ± 0.05	0.99 ± 0.043
FFD (*a* = 10 nm, near)	1.94 ± 0.05	3.05 ± 0.05	0.79 ± 0.026
FFD (*a* = 10 nm, far)	1.94 ± 0.05	3.73 ± 0.05	0.96 ± 0.012
FFD (*a* = 20 nm, near)	1.88 ± 0.25	2.76 ± 0.25	0.73 ± 0.075
FFD (*a* = 20 nm, far)	1.88 ± 0.25	3.55 ± 0.25	0.94 ± 0.059
Microtubule-like geometry (near)	5.17 ± 0.25	6.48 ± 0.25	0.63 ± 0.18
Microtubule-like geometry (far)	5.17±0.25	8.02 ± 0.25	0.76 ± 0.14

The deviation from unity indicates sublinear scaling with *N*. The error bar in the stall force data is estimated as half of the step size for force used in the simulations. For two filaments, ‘near’ refers to zero base separation and ‘far’ refers to base separation 10 times the radius of cross-section (except for the last, where the base separation is 150 nm).

## Discussion

Polymerisation-driven force generation by filaments has many biological applications, and the problem has been extensively studied experimentally as well as theoretically. A central quantity of interest here is the stall force of a bundle of *N* filaments, and its scaling behavior with *N*. Recent years have seen a spurt of activity in theoretical modeling in this field, but barring a few^14,18,41^, most of the models are one-dimensional in nature, and do not consider explicitly (three-dimensional) monomer diffusion in space^6–11^, or even the dynamics of the object (barrier) that is being pushed by the filaments^7–10^. Here, we have introduced and studied a more general model in which the effects of polymerisation-driven growth of the filament and the presence of the physical barrier on monomer concentration are included, and the consequent fall in the monomer adsorption rate is estimated. We showed that, in general, the physical barrier affects the monomer concentration profile, causing a drop in the growth rate in addition to being a steric hindrance to growth, when the filament tip and the barrier are within a distance of approximately 3–4 times the radius of cross-section of the filament tip (imagined as having a solid disc-like face). We then investigated, by mathematical analysis as well as Brownian dynamics simulations, how the collective dynamics of a bundle of filaments is affected by this barrier-induced hindrance to free diffusion. In the process, we also encountered diffusive interaction between filaments that naturally appears when nearby filaments grow together by diffusion-limited adsorption, but its effects are particularly noticeable in the presence of a barrier as the latter reduces the spread in length across different filaments and thereby forces the tips to be close to each other.

The mathematical part of our study uses a continuum Fokker-Planck equation to describe the collective growth of *N* identical filaments against a rigid barrier, the latter’s motion including drift towards the filaments due to an externally applied force, and random diffusive motion powered by thermal noise. We then use an adiabatic approximation where the stationary monomer density profile is assumed to respond instantly to changes in the positions of the filament tips and the barrier. The on-rate for adsorption of monomers onto a filament tip is calculated, and also measured directly in simulations. By assuming a simple analytical form for the boundary-affected reduced on-rate, consistent with observations, we studied steady state properties of the filament population. In particular, we derived analytical expressions for the mean filament growth velocity and the stall force of an assembly. These expressions involve the probability distribution for the filament tip-barrier separations (‘gaps’), which was calculated explicitly in a few special cases of interest. All analytical predictions were subjected to verification in Brownian dynamics simulations, which were also used to explore the consequences of having more complex microtubule-like multi-stranded structure for the filaments.

In this work, we established clearly that, in general, a physical barrier may be expected to cause reduction in the rate of growth of a polymer growing towards it, and this effect also reduces the stall force of the filament. However, as long as the filaments have sufficient lateral separation from each other, the stall force for *N* filaments increases linearly with *N*. Nonlinear scaling appears when the filaments are brought close together to form a bundle; in this case, diffusive interaction between the growing filament tips leads to a non-additive combined stall force of the bundle. The effects of this diffusive interaction are most visible in a multi-stranded filament like a microtubule; here, we show conclusively in simulations that the net stall force of the filament is much less than the sum of the stall forces of the individual protofilaments^21^. Similarly, the combined stall force for two microtubules is generally less than twice the stall force of one. Diffusive coupling, when significant, leads to sublinear scaling of stall force with the number of filaments, a notable prediction from our studies. Specifically, in microtubules, we report the existence of strong diffusive coupling between different protofilaments, arising by virtue of their tight packing, which leads to smaller combined stall force, compared to a hypothetical situation where each protofilament grows independent of the others. We also observe a remarkable phenomenon in our simulations; two microtubules, when growing against a *super-stall* force, stand their ground after retreating to a new ‘equilibrium’ position, and refuse to be continuously pushed backward unlike a single microtubule, or simpler (single-strand) flat-faced filaments. At the moment, we lack a clear understanding of the mechanism or the implications of this observation, and investigating it further is one of our immediate goals for the future.

Among the limitations of our study, we have treated diffusing monomers as point point particles devoid of size and shape; therefore, Brownian rotation of monomers and orientational constraints to their adsorption at the growing tip have been ignored (see, for instance, a recent computational study on the growth of actin bundles^41^ where finite size of binding monomers are consequent steric effects have been included). We do not believe that this will impact our principal conclusions, but if taken into account, could reduce the on-rate uniformly everywhere. Yet another important omission in our model, in the context of microtubules, is that we have not included GTP hydrolysis and the consequent dynamic instability. Recent theoretical work^10^ has shown that the combined stall force of a bundle of *N* microtubules with dynamic instability scales superlinearly with *N*. Bundle catastrophes, observed in microtubules growing close together^25^ seems to be a collective catastrophe phenomenon which could be studied further using the approach developed in this paper. Even within our model, the large *N* behaviour of the collective force produced by a filament bundle remains to be explored, both mathematically and computationally. In general, it would be interesting to see how the competition between diffusive coupling and dynamic instability, which appear to have opposite effects on the scaling of force with number, affects collective force generation and dynamic instability in a microtubule bundle.

## Electronic supplementary material

Supplementary Information
