# Supplementary material for: Polymerisation force of a rigid filament bundle: diffusive interaction leads to sublinear force-number scaling
Source: Sci Rep. 2018 Feb 6;8:2526. doi: 10.1038/s41598-018-20259-7 (PMC5802839; doi:10.1038/s41598-018-20259-7)
Supplement: Supplementary file 1 — Supplementary Information [file 41598_2018_20259_MOESM1_ESM.pdf]

Supplementary material to Polymerisation force of a rigid filament bundle: diffusive interaction leads to sublinear force-number scaling

Jemseena Valiyakath[a] and Manoj Gopalakrishnan

Department of Physics, Indian Institute of Technology, Madras, India

[a] Present address: International Centre for Theoretical Sciences, Tata Institute of Fundamental Research, Bangalore 560089, India

1 Modification of monomer binding rate by the wall

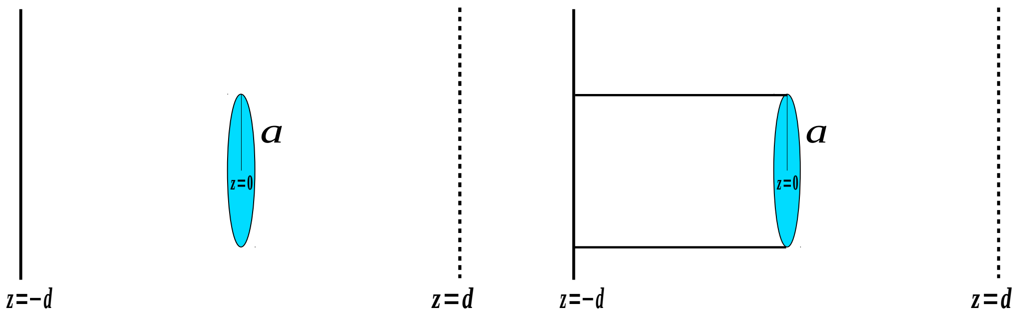

(a) (b)

Figure S1: (a)Schematic figure of the geometry used to solve Eq.S1. A thin circular disk of radius 'a' kept in between two rigid infinite walls, which acts as an absorbing region for the incoming particles. (b) Schematic figure of the geometry used in Brownian dynamic simulations. In addition to the conditions in (a), a reflecting boundary condition is imposed on the cylindrical wall.

Assuming the filament to be a linear chain of monomers, the probability that a monomer adds in the time interval dt is $k_{\rm on}(d)dt$, where $k_{\rm on}(d)$ is the rate at which monomers are added to the tip. In our model, $k_{\rm on}(d)$ in general depends on the separation between the filament tip and the barrier, denoted as d. When the filament tip is far away from the barrier, $k_{\rm on}(d) \to k_{\rm on}(\infty)$, equivalent to the case studied earlier[1-4]. Due to the steric hindrance arising out of the presence of the wall, a monomer can be added only if there exists a sufficient space between the filament tip and the barrier, equal to the size of the monomer.

Assuming steady state conditions, the monomer concentration $C(\mathbf{r}, t)$ satisfies Laplace's equation

$$D\nabla^2 C(\mathbf{r}) = 0, \tag{S1}$$

where $\mathbf{r}$ is the position measured with respect to the centre of the surface and D is the diffusion coefficient of monomers. In diffusion-limited growth, the steady state adsorption rate of monomers to a surface S is given by the integral

$$k_{\rm on} = D\int_S \nabla C \cdot d\mathbf{S}. \tag{S2}$$

To find $C(\mathbf{r})$, we solved Eq.S1 in a geometry (having cylindrical symmetry) as shown in Fig.S1a, consisting of an absorbing disk of radius a and zero thickness placed at $z = 0$ and an infinite reflecting

boundary at $z = d$. Given this geometry, the solution of Eq.S1, denoted as $C_d(\mathbf{r})$, is given by

$$C_d(\rho, z) = \int_0^\infty \alpha_k J_0(k\rho)\ e^{-kz}[1 + e^{-2k(d-z)}]dk \qquad z > 0 \tag{S3}$$

$$C_d(\rho, z) = \int_0^\infty \beta_k J_0(k\rho)\ e^{kz}[e^{2kd} + e^{-2kz}]dk \qquad z < 0, \tag{S4}$$

where α_k and β_k are constants to be fixed and the subscript d indicates the location of the barrier. To see how the presence of the reflecting wall at $z = d$ affects the on-rate of particles coming from $z > 0$, we evaluate the integral in Eq.S2 using Eq.S3. The constant α_k in Eq.S3 is fixed such that $C_d(\rho, z)$ in the positive z region satisfies the full set of boundary conditions:

$$\begin{aligned} C_d(\rho, z = 0^+) &= 0 \qquad & 0 \leq \rho \leq a \\ C_d(\rho, z) &= C_0 \qquad & \rho \gg a \\ \left.\frac{\partial C_d(\rho, z)}{\partial z}\right|_{z=d} &= 0 \qquad & 0 \leq \rho \leq \infty. \end{aligned} \tag{S5}$$

Consistent with the above boundary conditions, the solution in the region $z > 0$ becomes,

$$C_d(\rho, z) = C_0 - \frac{2C_0}{\pi}\int_0^\infty dk\ \frac{\sin(ka)}{k}\ J_0(k\rho)\ e^{-kz}\left[\frac{1 + e^{-2k(d-z)}}{1 + e^{-2kd}}\right]. \tag{S6}$$

As a special case, for $z = d$, the solution, obtained after performing the integration in Eq.S6 is

$$C_d(\rho, d) = C_0 - \frac{4C_0}{\pi}\sum_{n=0}^{\infty}(-1)^n \sin^{-1}\left[\frac{2a}{\rho_+ + \rho_-}\right], \tag{S7}$$

where

$$\rho_+ = \sqrt{(\rho + a)^2 + (2n + 1)^2 d^2}; \qquad \rho_- = \sqrt{(\rho - a)^2 + (2n + 1)^2 d^2}. \tag{S8}$$

For comparison, if the wall were not present, the corresponding solution (again, at $z = d$) would be

$$C_\infty(\rho, d) = C_0 - \frac{2C_0}{\pi}\sin^{-1}\left[\frac{2a}{\sqrt{(\rho + a)^2 + d^2} + \sqrt{(\rho - a)^2 + d^2}}\right]. \tag{S9}$$

In Fig.S2, we show the concentration profile (dimensionless, scaled using the asymptotic value C_0) in the radial direction, given by Eq.S7 and Eq.S9, for $a = 20$ nm and $d = 10$ nm. The figure shows that the presence of the wall enhances monomer depletion in front of the growing filament tip, and this effect is found over a (radial) distance nearly 4 times the radius of cross-section of the absorbing disk. It is natural to expect that this depletion will also cause a fall in the rate of adsorption of the monomers at the disk, which we calculate next using Eq.S2.

$$k_{\text{on}}(d) = -D\int_0^a 2\pi\rho d\rho \left.\frac{\partial C_d(\rho, z)}{\partial z}\right|_{z=0^+}. \tag{S10}$$

Using the expression for $C_d(\rho, z)$ in the region $z > 0$ given by Eq.S6, we have

$$\left.\frac{\partial C_d(\rho, z)}{\partial z}\right|_{z=0^+} = -D\frac{2C_0}{\pi}\int_0^\infty dk\ \sin(ka)\ J_0(k\rho)\left[\frac{1 - e^{-2kd}}{1 + e^{-2kd}}\right]. \tag{S11}$$

Substituting Eq.S11 in Eq.S10 and performing the integration, we find

$$k_{\text{on}}(d) = 4C_0 Da\left[1 - 2\sum_{n=0}^{\infty}(-1)^n \frac{a - \sqrt{a^2 - l^2}}{a}\right]. \tag{S12}$$

with

$$l^2 = \frac{1}{4}\left[\sqrt{4a^2 + 4(n + 1)^2 d^2} - \sqrt{4(n + 1)^2 d^2}\right]^2. \tag{S13}$$

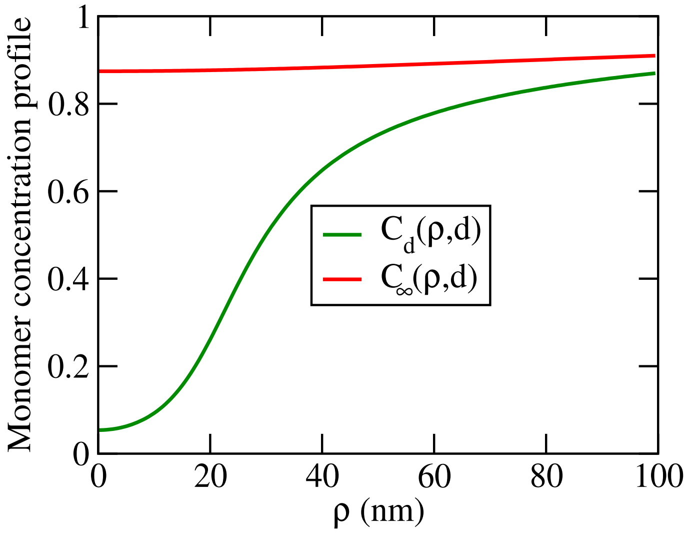

Figure S2: A comparison of the concentration profile of free monomers along the radial direction, given by Eq.S7, and Eq.S9, for disk radius $a = 20$ nm at $z = 10$ nm, when the reflecting barrier is placed at $z = 10$ nm (green) and $z = \infty$ (red). Far away from the barrier, the concentration is given by the asymptotic value C_0. Note that the presence of the barrier enhances depletion of monomers in front of the absorbing disk.

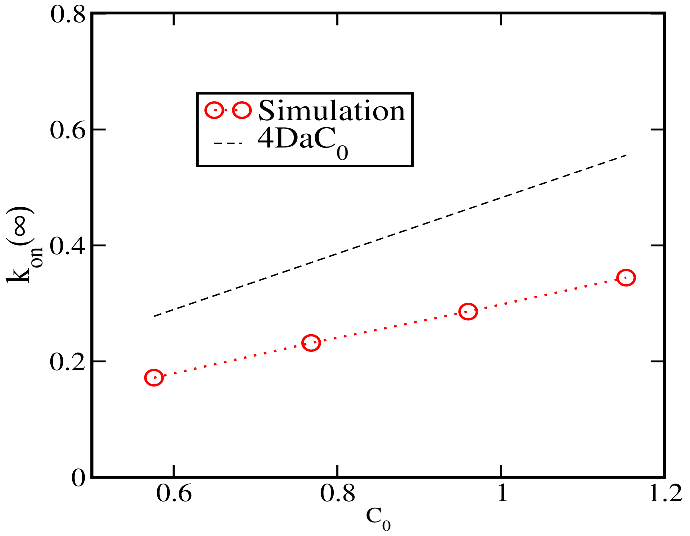

Figure S3: A comparison of the asymptotic value ($d \gg a$) of on-rate, $k_{on}(\infty)$ obtained from Brownian dynamics simulation with the theoretical expression, for $R = 10$ nm.

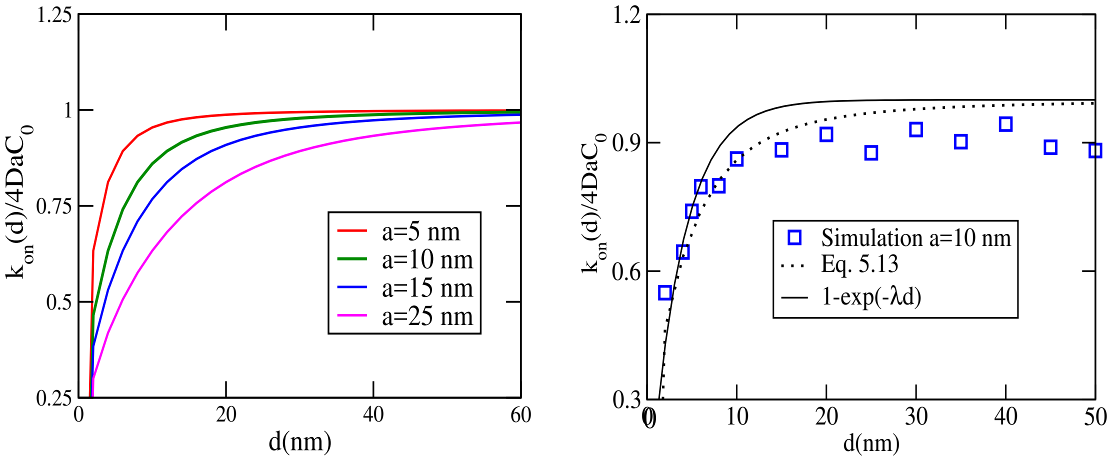

(a) (b)

Figure S4: (a) On-rate of particles to a circular disk of radius 'a' as a function of distance between the disk and the reflecting wall for different disk radii, given by Eq.S12. (b) A comparison of analytical result for the on-rate given by Eq.S12 with results from Brownian dynamics simulations. The Brownian dynamics simulations are carried out for a geometry shown in Fig. S1b; for more discussions on details of simulations, see the main text. The thick line is shown for the approximated scaled expression of the on-rate, $k_{\rm on}(d)/k_{\rm on(\infty)} = 1 - \exp(-\lambda d)$ with the best fit value of $\lambda = 0.275$ nm^{-1}. The dotted line is the analytical expression given by Eq.S12.

In the limit $d \to \infty$ (disk far away from the barrier), the quantity l^2 given by Eq.S13 goes to zero and the on-rate takes the simple expression [5],

$$\lim_{d\to\infty} k_{\rm on}(d) \equiv k_{\rm on}(\infty) = 4DC_0 a, \tag{S14}$$

as expected. Comparison of $k_{\rm on}(\infty)$ obtained from Brownian dynamics simulation with Eq.S14, for various C_0 is shown in Fig. S3.

The on-rate decays monotonically as the wall-disk separation decreases, suggesting that the presence of a barrier decreases the likelihood of particles being getting trapped and hence slows down the growth rate, see Fig.S4. We also verified the prediction for on-rate given by Eq.S12, by doing Brownian dynamics simulations, for more details see section on simulations in the main text. A visual inspection of Fig. S4 suggests that boundary induced drop in on-rate comes into play when the separation between the barrier and radius of cross section of the absorbing disk are comparable , i.e., $d \sim a$. Unfortunately, the expression for the flux given in Eq. S12 is not simple enough to be used directly for further mathematical calculations, hence we approximate Eq. S12 by the simpler form: $k_{\rm on} \sim k_{\rm on}(\infty)(1 - e^{-\lambda d})$. The inverse of the parameter λ gives a measure of the size of depletion zone, i.e., $\lambda \sim 1/a$. In Fig.S4b, we give a fit of the approximate expression for on-rate with the simulation data for $a = 10$ nm. The best fit parameter value of λ in this case is 0.275 nm^{-1}.

2 $1/\lambda$ expansion for $V_N(f)$ and f_s^N:

As a simple extension of the calculation discussed for the case $\lambda = \infty$ (see main text), an asymptotic expansion of the expressions for average filament velocity and stall force in the large λ limit is carried out, to quantify small deviations from the constant on-rate case. Consider the equation for single filament gap distribution, $\phi_N(y)$ given by Eq.20 in the maint text. Performing an integration with respect to y in Eq.20, and using Eq.39 and Eq.40 (see equations after this section) we get,

$$\langle V(y)\rangle - V_w - [D(0) + D_w]\phi_N(0) - (N-1)D_w\phi_N(0) = 0, \tag{S15}$$

which yields

$$\phi_N(0) = \frac{\langle V(y)\rangle - V_w}{D(0) + ND_w}. \tag{S16}$$

Substituting Eq.S16 in the expression for $V_N(f)$ given by Eq.13 in the main text gives

$$V_N(f) = \frac{V_w D(0) + N D_w \langle V(y)\rangle}{D(0) + N D_w}. \tag{S17}$$

In general, $\phi_N(y)$ is a function of λ as well, therefore let us express $\phi_N(y)$ explicitly as a function of λ and N i.e.,

$$\phi_N(y) \equiv \phi(y; N, \lambda). \tag{S18}$$

The quantity $\langle V(y)\rangle$ is calculated using Eq.25 in the main text,

$$\langle V(y)\rangle = V_0 - V_1 \int_0^\infty e^{-\lambda y} \phi(y; N, \lambda) dy. \tag{S19}$$

Next, we expand $\phi(y; N, \lambda)$ in powers of λ:

$$\phi(y; N, \lambda) = \phi^0(y; N) + \frac{1}{\lambda}\phi^1(y; N) + \mathcal{O}(\frac{1}{\lambda^2})... \tag{S20}$$

From Eq. 38 of the main text, we have

$$\phi^0(y; N) = \Delta_2 e^{-\Delta_2 y}, \tag{S21}$$

where Δ_2 is given by Eq35 (main text). To evaluate $\langle V(y)\rangle$, we substitute Eq.S20 in the integral given in Eq.S19, also substitute $\phi^0(y; N)$ from equation Eq.S21, and we find

$$\langle V(y)\rangle \approx V_0 - \frac{V_1 \Delta_2}{\lambda} + \mathcal{O}(\frac{1}{\lambda^2}). \tag{S22}$$

Therefore, from Eq.S17, we have

$$V_N(f) \approx \frac{V_w D_0 + N D_w V_0}{D_0 + N D_w}\left[1 - \frac{1}{\lambda}\frac{V_1(V_0 - V_w)}{D_0 + N D_w} + \mathcal{O}(\frac{1}{\lambda^2})\right]. \tag{S23}$$

The stall force is obtained from Eq.S23 using the defining relation $V_N(f_s^N) = 0$, and takes the form of an power-series expansion in $1/\lambda$:

$$f_s^N \approx \frac{2N k_B T}{\delta}\left(\frac{k_{\rm on}(\infty) - k_{\rm off}}{k_{\rm on}(\infty) + k_{\rm off}}\right)\left[1 - \frac{2}{\lambda\delta}\frac{k_{\rm on}(\infty)}{[k_{\rm on}(\infty) + k_{\rm off}]} + \mathcal{O}(\frac{1}{\lambda^2})\right]. \tag{S24}$$

From Eq.S23 and Eq.S24, it is evident that the barrier-induced inhibition of free diffusion causes a drop in the mean velocity and stall force, while the linear scaling of stall force with the number of filaments holds, at least to first order in $1/\lambda$. Numerical simulations, discussed in the main text, indicate that this result holds for arbitrary λ, under conditions where the filaments grow independent of each other.

3 Gap distribution for $N = 2$

Equation for $F_2(y)$

From Eq.19 in the main text, we have a set of N equations. For $i = 1$ we have

$$[V(y_1) - V_w]\Phi(Y) + \frac{\partial}{\partial y_1}[D(y_1)\Phi(Y)] + D_w \sum_{j=1}^{N} \frac{\partial}{\partial y_j}\Phi(Y) = 0. \tag{S25}$$

Similarly, for $i = 2$,

$$[V(y_2) - V_w]\Phi(Y) + \frac{\partial}{\partial y_2}[D(y_2)\Phi(Y)] + D_w \sum_{j=1}^{N} \frac{\partial}{\partial y_j}\Phi(Y) = 0. \tag{S26}$$

Eq. S25 and S26 can be rewritten as follows

$$[V(y_1) - V_w]\Phi(Y) + \frac{\partial}{\partial y_1}[D_w + D(y_1)]\Phi(Y) + D_w \frac{\partial}{\partial y_2}\Phi(Y) + D_w \sum_{j=3}^{N} \frac{\partial}{\partial y_j}\Phi(Y) = 0. \tag{S27}$$

$$[V(y_2)-V_w]\Phi(Y)+\frac{\partial}{\partial y_2}[D_w+D(y_2)]\Phi(Y)+D_w\frac{\partial}{\partial y_1}\Phi(Y)+D_w\sum_{j=3}^{N}\frac{\partial}{\partial y_j}\Phi(Y)=0. \tag{S28}$$

Let

$$\psi(y_1,y_2)=\int\Phi(y_1,y_2,y_3,..y_N)dy_3,....,dy_N. \tag{S29}$$

Now performing the integration given by Eq.S29 in Eq.S27 and S28 we get,

$$[V(y_1)-V_w]\psi(y_1,y_2)+\frac{\partial}{\partial y_1}[D_w+D(y_1)]\psi(y_1,y_2)]+D_w\frac{\partial}{\partial y_2}\psi(y_1,y_2)=(N-2)D_w\xi(y_1,y_2), \tag{S30}$$

$$[V(y_2)-V_w]\psi(y_1,y_2)+\frac{\partial}{\partial y_2}[D_w+D(y_2)]\psi(y_1,y_2)+D_w\frac{\partial}{\partial y_1}\psi(y_1,y_2)=(N-2)D_w\xi(y_1,y_2), \tag{S31}$$

where

$$\xi(y_1,y_2)=\int\Phi(y_1,y_2,y_3=0,y_4..y_N)dy_4..dy_N. \tag{S32}$$

For $N=2$, Eq.S30 and S31 become a self-contained set of equations:

$$[V(y_1)-V_w]\psi(y_1,y_2)+\frac{\partial}{\partial y_1}[D(y_1)+D_w]\psi(y_1,y_2)+D_w\frac{\partial}{\partial y_2}\psi(y_1,y_2)=0, \tag{S33}$$

$$[V(y_2)-V_w]\psi(y_1,y_2)+\frac{\partial}{\partial y_2}[D(y_2)+D_w]\psi(y_1,y_2)]+D_w\frac{\partial}{\partial y_1}\psi(y_1,y_2)=0. \tag{S34}$$

Using Eq.S33 and S34, and using the definition (see also Eq.21 in main text),

$$F_2(y)=\psi(y,y_2=0), \tag{S35}$$

we arrive at the following equation for $F_2(y)$:

$$\frac{d}{dy}\Bigg[D_wD(0)+D(y)[D_w+D(0)]\Bigg]F_2(y)=$$
$$\Bigg[[D_w+D(0)][V_w-V(y)-D'(y)]-D_w[V_w-V(0)-D'(0)]\Bigg]\times F_2(y). \tag{S36}$$

Eq.S36 is a first order homogeneous differential equation, whose solution is given by

$$F_2(y)=\mathcal{B}\exp\Bigg(\int^y\eta(y')dy'\Bigg), \tag{S37}$$

with

$$\eta(y)=\frac{\Bigg[[D_w+D(0)][V_w-V(y)-D'(y)]-D_w[V_w-V(0)-D'(0)]\Bigg]}{\Bigg[D_wD(0)+D(y)[D_w+D(0)]\Bigg]}. \tag{S38}$$

Fixing of constants $\mathcal{A}$ and $\mathcal{B}$

From the definition of $\phi_N(y)$ we have

$$\int_0^\infty\phi_N(y)dy=1, \tag{S39}$$

also from the definition of $F_N(y)$

$$\int_0^\infty F_N(y)dy=\phi_N(y=0). \tag{S40}$$

To fix the unknowns $\mathcal{A}$ and $\mathcal{B}$ which appear respectively in Eq.23 in the main text and Eq.S37, we use the two conditions given by Eq.S39 and Eq.S40, Now substituting the forms for $V(y)$ and $D(y)$ from Eq.25 in the main text and performing the integration in Eq.S37, we get

$$F_2(y) = \frac{\mathcal{B}}{k_2} \exp\left\{P_1 \ln\left[\frac{k_1}{k_2} - e^{-\lambda y}\right]\right\} e^{Q_1 y}, \tag{S41}$$

where,

$$\begin{aligned} P_1 &= \frac{D_w[V(0)+D'(0)] + D(0)V_w - [D_w + D(0)]V_0}{\lambda k_1} + \frac{[D_w + D(0)]V_1}{\lambda k_2} - 1, \\ Q_1 &= \frac{D_w[V(0)+D'(0)] + D(0)V_w - [D_w + D(0)]V_0}{k_1}, \\ k_1 &= D(0)D_w + [D_w + D(0)]D_0; \quad k2 = [D_w + D(0)]D_1. \end{aligned} \tag{S42}$$

Substituting Eq.S41 in Eq.23 in the main text, with $N = 2$, and carrying out the integration we get,

$$\phi_2(y) = \frac{\mathcal{D}^{P_2}}{D_1} e^{-Q_2 y}\left[1 - \mathcal{D}e^{-\lambda y}\right]^{-P_2} \times \left\{\mathcal{A} + \mathcal{B}\frac{D_w \mathit{\Omega}^{-P_1}}{k_2 \mathcal{D}^{P_2-1}} \sum_{m=0}^{\infty}\sum_{n=0}^{\infty} \frac{(1-P_2)_m(-P_1)_n}{m!n!} \mathcal{D}^m \mathit{\Omega}^n \frac{e^{(Q_1+Q_2)y} e^{-(m+n)\lambda y}}{[Q_1 + Q_2 - (m+n)\lambda]}\right\}. \tag{S43}$$

Also, using the condition that $\int_0^\infty F_2(y)dy = \phi_2(y = 0)$, we find

$$\mathcal{A} = \gamma \mathcal{B}$$

where

$$\begin{aligned} \gamma = &-\frac{D_w \mathit{\Omega}^{-P_1}}{k_2 \mathcal{D}^{P_2-1}} \sum_{m=0}^{\infty}\sum_{n=0}^{\infty} \frac{(1-P_2)_m(-P_1)_n}{m!n!} \mathcal{D}^m \mathit{\Omega}^n \frac{1}{[Q_1 + Q_2 - (m+n)\lambda]} \\ &- \frac{D_1 \mathit{\Omega}^{-P_1}}{k_2 \mathcal{D}^{P_2}} [1 - \mathcal{D}]^{P_2} \sum_{n=0}^{\infty} \frac{(-P_1)_n}{n!} \mathit{\Omega}^n \frac{1}{[Q_1 - n\lambda]}. \end{aligned} \tag{S44}$$

The expression for $\mathcal{B}$ is determined using normalization condition for $\phi_2(y)$:

$$\begin{aligned} 1 = \mathcal{B}\Bigg\{ &\frac{\gamma \mathcal{D}^{P_2}}{D_1} \sum_{n=0}^{\infty} \frac{(P_2)_n}{n!} \mathcal{D}^n \frac{1}{[Q_2 + n\lambda]} + \frac{D_w}{D' k_2 \mathit{\Omega}^{P_1}} \times \\ &\sum_{l=0}^{\infty}\sum_{m=0}^{\infty}\sum_{n=0}^{\infty} \frac{(P_2)_l}{l!} \frac{(1-P_2)_m}{m!} \frac{(-P_1)_n}{n!} \mathcal{D}^{l+m} \mathit{\Omega}^n \frac{1}{[(n+m+l)\lambda - Q_1][Q_2 + Q_1 - (n+m)\lambda]}\Bigg\}. \end{aligned} \tag{S45}$$

4 References

[1]Peskin, C. S., G. M. Odell, and G. F. Oster, 1993. Cellular motions and thermal fluctuations: the Brownian ratchet. Biophys. J 65:316–324.
[2]Mogilner, A., and G. Oster, 1999. The polymerization ratchet model explains the force-velocity relation for growing microtubules. Eur. Biophys. J. 28:235–242.
[3] van Doorn, G. S., C. Tanase, B. M. Mulder, , and M. Dogterom, 2000. On the stall force for growing microtubules. Eur. Biophys. J. 29:2–6.
[4] Das, D., D. Das, and R. Padinhateeri, 2014. Collective force generated by multiple biofilaments can exceed the sum of forces due to individual ones. New J. Phys. 16:063032.
[5] Crank, J., 1975. The Mathematics of Diffusion. Oxford.
